# Further evidence for the association of CYP2D6*4 gene polymorphism with Parkinson’s disease: a case control study

**DOI:** 10.1186/s41021-017-0078-8

**Published:** 2017-07-01

**Authors:** Anwarullah, Muhammad Aslam, Mazhar Badshah, Rashda Abbasi, Aneesa Sultan, Kafaitullah Khan, Nafees Ahmad, Jakob von Engelhardt

**Affiliations:** 1 Synaptic Signalling and Neurodegeneration, German Center for Neurodegenerative Diseases (DZNE), Bonn, Germany; 2 Institute of Pathophysiology, University Medical Center of the Johannes Gutenberg University Mainz, Duesbergweg 6, 55128 Mainz, Germany; 3 Department of Biochemistry, Quaid-i-Azam University, Islamabad, Pakistan; 4 Institute of Biomedical and Genetic Engineering (IBGE), Islamabad, Pakistan; 5 Department of Neurology, Shaheed Zulfiqar Ali Bhutto Medical University, Islamabad, Pakistan; 6 Department of Zoology, University of Balochistan, Quetta, Pakistan

**Keywords:** *CYP2D6* gene, Poor metabolizer status, Parkinson’s disease

## Abstract

**Background:**

Genetic and environmental risk factors play an important role for the susceptibility to sporadic Parkinson’s disease (PD). It was hypothesized that a splice variant of the *CYP2D6* gene (CYP2D6*4 allele) is associated with PD because it alters the ability to metabolize toxins and in particular neurotoxins. *CYP2D6* codes for the drug metabolizing enzyme debrisoquine 4-hydroxylase. The CYP2D6*4 variant results in an undetectable enzyme activity and consequently in a reduction in metabolism of some toxins.

**Methods:**

Some of agricultural chemicals have neurotoxic potential and CYP2D6 is involved in their detoxification. Thus, we conducted a case control study to investigate the association of the CYP2D6*4 with PD in a Pakistani subpopulation that is known to be exposed to high levels of some agricultural pesticides, insecticides and herbicides.

**Results:**

We found a significantly higher allele and genotype frequency of the CYP2D6*4 variant in 174 sporadic PD patients when compared to 200 controls. In addition, there was a trend to an earlier age of PD onset and a tremor dominant phenotype in CYP2D6*4 variant carriers.

**Conclusion:**

Our data provide further evidence that a poor metabolizer status may increase the risk to develop PD especially in populations that are exposed to environmental toxins.

## Background

Parkinson’s disease (PD) is a slowly progressing neurodegenerative disorder primarily affecting motor function although nonmotor symptoms such as autonomic, cognitive, and psychiatric manifestations may also appear throughout the disease course [1]. A positive family history is associated with a high risk of PD in many populations worldwide suggesting a role of genetic factors. Indeed several causative genes have been identified so far through genetic analysis in rare multicase families with Mendelian inheritance of PD. The etiology of more common sporadic idiopathic Parkinson’s disease (PD) is however not known, but is thought to be multifactorial with environmental factors and aging increasing the risk in genetically predisposed individuals [2]. Several studies hypothesized that individuals with certain genotypes of drug metabolizing enzymes may have difficulty in metabolizing one or more environmental neurotoxins and that this “poor metabolizer” status could make them susceptible to developing PD following exposure to such toxins [3].

Enzymes encoded by the cytochrome P450 gene family (CYP enzymes) represent 70–80% of phase I metabolism and are responsible for the biotransformation of exogenous drugs and toxins to polar metabolites, which can be excreted by the kidneys [4, 5]. The CYP enzyme debrisoquine 4-hydroxylase encoded by the *CYP2D6* gene (MIM: 124030) is of particular importance in the context of PD since it is expressed in neurons and metabolizes endogenous and environmental neurotoxins that have been associated with idiopathic PD [6] Importantly, individuals with PD have lower CYP2D6 expression in the brain [7].

A single nucleotide polymorphism (rs3892097; c.1846G > A) in the *CYP2D6* gene results in a functionally deficient variant of the enzyme (the so called CYP2D6*4 isoform). Currently it is believed that this is a frequent *CYP2D6* variant leading to a deficiency in enzyme activity and it is believed to result in a poor metabolizer status. Moreover, the CYP2D6*4 variant has been considered as a genetic susceptibility factor in idiopathic PD, consistent with the hypothesis that a poor metabolizer status increases the disease risk. Case–control association studies have been performed in many populations to test this association [8–10]. The association between CYP2D6*4 and PD was confirmed in some European populations where CYP2D6*4 allelic frequency is relatively higher compared to Asian populations [11].

Interestingly, postmortem toxicology and epidemiological studies found some organophosphate and organochlorine pesticides as an environmental risk factor for PD [12–18]. Brown et al., [19] extensively reviewed the potential neurotoxic mechanisms by which certain pesticide chemicals may influence PD risk. In vitro studies indicated that CYP2D6 at least in part participate in the metabolism of some organophosphate and organochlorine pesticides (such as Parathion, Chlorpyrifos and Diazinon) [20–23]. A combined effect of a higher exposure to potentially toxic pesticides and a reduced CYP2D6 enzyme activity due to CYP2D6*4 allele therefore represents a potential risk factor for PD. Indeed a previous study indicated that the effect of CYP2D6*4 allele on the risk of PD increases with higher levels of pesticide exposure [24].

Extensive and indiscriminate use of agricultural chemicals in Pakistan and environmental exposure of some Pakistani population groups to organophosphate and organochlorine pesticides has been reported previously [25–28]. We therefore investigated the association of CYP2D6*4 allelic variant with PD susceptibility in a Pakistani population subgroup. by genotyping 174 PD patients and 200 controls with comparable pesticide exposure., We found a significantly higher allelic and genotype frequency of the CYP2D6*4 variant in PD patients when compared to controls.

## Methods

### Enrollment and sampling

Medical records of 174 patients with primary diagnosis of idiopathic PD were collected from the neurology department of Shaheed Zulfiqar Ali Bhutto Medical University Islamabad. The study was performed in accordance with the Declaration of Helsinki and was approved by the Research Ethics Committee of the Institute of Biomedical and Genetic Engineering (IBGE) Islamabad, Pakistan. Written informed consent was obtained from all subjects. A total of 5–10 ml blood was drawn from all study participants. The control individuals (*n* = 200) were matched with the cases according to age at collection (Cases: 59.1 ± 8.9 years; controls: 60.4 ± 8.8 years), ethnicity (Pathan/Khyber Pakhtunkhwa province of Pakistan), gender (male to female ratio 2.3) and life style (rural farmland living). Genomic DNA was extracted with standard phenol chloroform method [29] and stored at −80° centigrade.

All PD patients were subjected to a detailed interview designed to obtain demographic and clinical information. The Unified Parkinson’s Disease Rating Scale (UPDRS) score was recorded for most patients (*n* = 149). The tremor dominant (TD) and postural instability/gait difficulty (PIGD) phenotypes of Parkinson’s patients were identified as described previously [30] Briefly, a ratio was obtained by dividing the sum of UPDRS “tremor items” 16, 20 and 21 by the sum of “postural instability and gait difficulty items” 13–15, 29 and 30. The cutoff scores of ≥1.5 and ≤1.0 were used for TD or PIGD phenotypes, respectively.

### *CYP2D6* (rs3892097; c.1846G > A) genotyping

Genotyping was performed as described previously for the single nucleotide polymorphism rs3892097 at the junction of exon 3 and 4 of the *CYP2D6* gene (c.1846G > A, also known as 1934G > A in the literature) [31]. Briefly, for each sample a 1.8 kb DNA fragment harboring rs3892097 at position 42128945 (GRCh38/hg19) of the human chromosome 22 was amplified by PCR in 20 μl reaction volume including 1 μl of 100 ng/μl genomic DNA, 12.12 μl PCR grade water, 2ul NH_4_ buffer (10X), 1.25 μl MgCl_2_ (25 m M), 1 μl dNTPs (2.5 mM), 0.25 μl Taq DNA polymerase and 1.5ul each forward and reverse primers. PCR was performed with 95 °C for 3 min, followed by 30 cycles of 94 °C for 30 s, annealing at 64 °C for 30 s and 72 °C for 60 s, and finally an extension at 72 °C for 5 min. The 1.8 kb fragment from the initial PCR was gel purified and used as template in allele specific PCR using the forward primer from the initial PCR and unique reverse primers for major and minor alleles. The product of the allele specific PCR (577 bp) was mixed with loading dye and subjected to prestained 2% agarose gel electrophoresis. The reproducibility of the genotyping methods was confirmed with bidirectional Sanger sequencing performed on randomly picked PD case and control samples (*n* = 20 per group) using M13 tailed primers flanking the rs3892097. The sequence of primers used for Sanger sequencing is as follows (*CYP2D6* specific sequence underlined):

- Forward sequencing primer TGTAAAACGACGGCCAGTATCTCTGACGTGGATAGGAGGT
- Reverse sequencing primer CAGGAAACAGCTATGACCTGATGGGCAGAAGGGCACAA

### Statistical analysis

Statistical Package for the Social Sciences (SPSS Version 13.0, Chicago, IL) was used for the statistical analysis. Goodness-of-fit Chi square method was used to test Hardy-Weinberg equilibrium. Pearson’s chi-square statistics was used as test for an association under allele contrast, recessive and dominant genetic models. Odds ratio (OR) and corresponding 95% CIs were calculated as a measure of association between PD risk and CYP2D6*4 polymorphism. Data are presented as mean ± standard deviation (SD). Significant association was assumed with a *p*-value < 0.05.

## Results

### Demographic and clinical characteristics of study subjects

We recruited PD patients (*n* = 174) who visited a single study center during a period of three years. The age of PD onset was 55 ± 13.0 years and the mean total UPDRS score was 87.8 (±32.5). Twenty-nine of the PD patients (19%) had a tremor dominant PD based on criteria described earlier [30] (see Methods). Patients and control individuals were similar according to their socioeconomic background (e.g., rurality and occupational structure) and were recruited during the same period. The aim was to recruit patients and controls that are comparably exposed to potential toxins such as pesticides.

### Association analysis

**Fig. 1:**
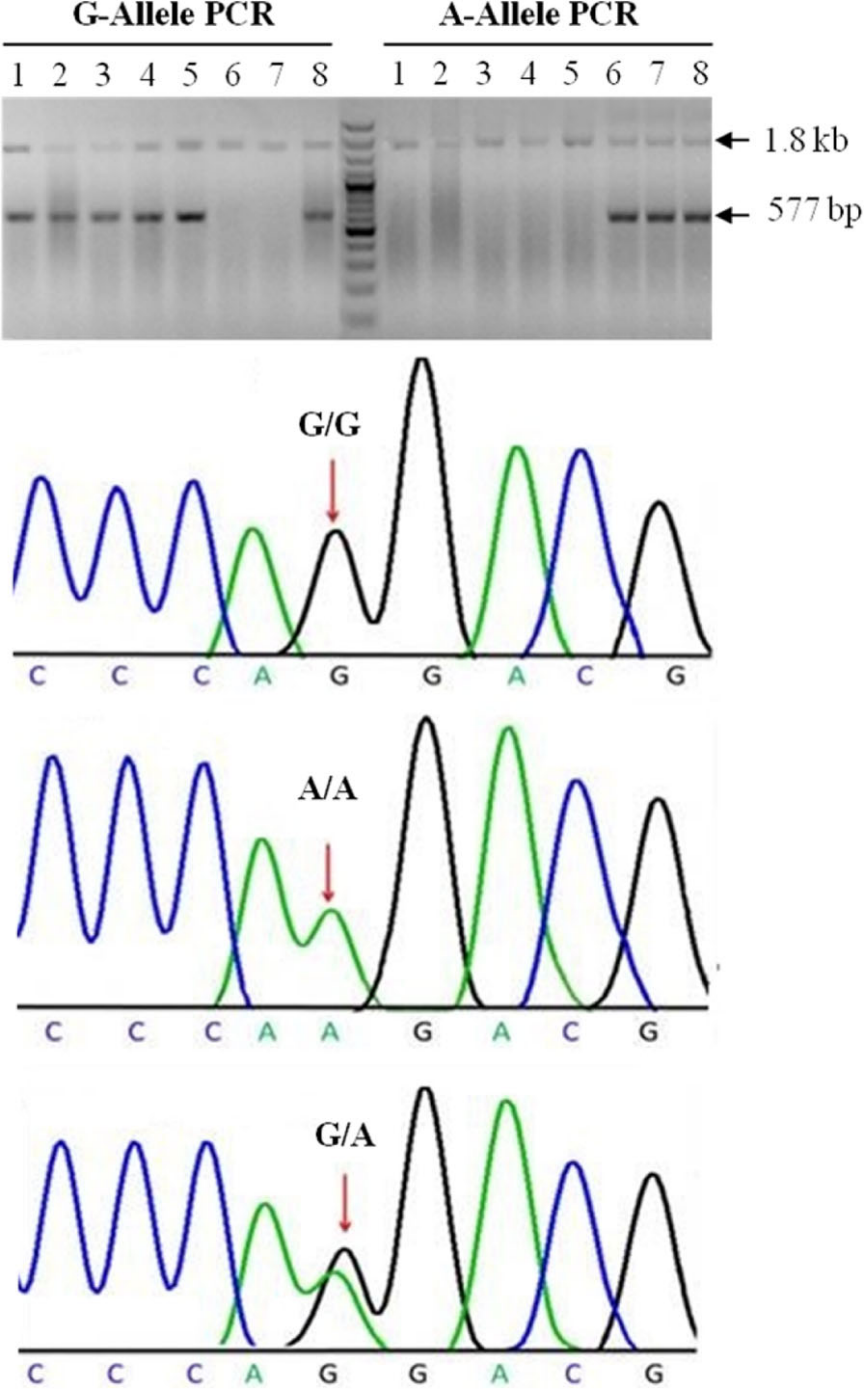
Genotyping of the CYP2D6 rs3892097 (G/A) polymorphism by an allele-specific PCR assay and validation of genotypes by Sanger sequencing. For each sample, two PCR reactions were performed using a 1.8 kb fragment harboring rs3892097 (G/A) polymorphism as template, each including a common forward primer and allele specific reverse primers (G-Allele PCR and A-Allele PCR). A 577-bp DNA fragment indicated the presence of the G or A allele. Individuals 1 to 5 are homozygous for the G-allele, individual 6 and 7 are homozygous for the A-allele and individual 8 is heterozygous. Sanger sequencing performed on individuals with different genotypes are shown below

Using allele specific PCR, we obtained genotypes for the SNP rs3892097 (G/A) for all sporadic idiopathic PD cases and control samples. To validate the method, we confirmed genotype results of 20 A-allele and 20 G-allele carriers with Sanger sequencing. A representative gel image and Sanger sequencing chromatograms for individuals homozygous for major allele (GG), homozygous for the risk allele (AA) and heterozygous (GA) are shown in Fig. 1. Allele and genotype frequencies and association analysis results are shown in Table 1. No deviation of genotype distribution from Hardy-Weinberg equilibrium was found in the control group. The frequency of the A-allele (coding for the CYP2D6*4 variant) in our control group was 4.4%. Importantly, there was a significant difference in the frequencies of the risk-associated allele (A) and genotype (GA and/or AA) between PD cases and controls (Table 1). The A-allele coding for the functionally deficient form of CYP2D6 enzyme (CYP2D6*4) increased the risk for PD more than two fold (OR: 2.52; 95% CI: 1.40 – 4.52; *p* = 0.001). A comparison of the genotype frequencies under a dominant model (GA + AA vs. GG) also indicated an increase in the risk of PD (OR = 2.02; 95% CI = 1.07-3.78; *p* = 0.02). We did not observe the AA-genotype in the control group. Therefore the OR cannot be calculated for a recessive genetic model (GG + GA vs. AA). The OR for the recessive model is indefinitely large since that there was no AA genotype in the control group. Importantly, the CYP2D6*4 carriers had a younger age of onset (53.0 ± 12.8) when compared to non-carriers (58.1 ± 10.2). The age of onset for PD in homozygous CYP2D6*4 carriers was even more reduced (45.8 ± 16.6)*.* Furthermore, 13% (4 out of 29) of the patients with CYP2D6*4 variant (A-allele) presented with a tremor dominant disease compared to 5% of non-carriers (6 out of 120).

**Table 1:** Genotype distribution, allele frequencies and association analysis of the rs3892097 (G/A) polymorphism in Pakistani population

Genotype distribution					
	GG	GA	AA	Total	HWE (*p*-value)
PD	145 (83.3%)	21 (12.0%)	8 (4.5%)	174	0.89
Control	182 (91.0%)	18 (9.0%)	0 (0%)	200	0.95
Total	327	39	8	374	0.92
Allelic distribution					
	G	A	Total		
PD	311 (89.3%)	37 (10.6%)	348		
Control	382 (95.5%)	18 (4.4%)	400		
Total	694	55	748		
Genetic model and statistical data					
Genetic model	Odds ratio (95% CI)	Chi-square	Degrees of freedom	*P*	
Allele contrast (G vs. A)	2.52 (1.40-4.52)	10.27	1	0.001	
Dominant (GA + AA vs. GG)	2.02 (1.07-3.78)	4.97	1	0.02	
Recessive (AA vs GG + GA)	Infinite^a^	9.23	1	0.002	

^a^The OR and 95% CI of the recessive model could not be calculated since there was no homozygous CYP2D6*4 observed in the control group

## Discussion

In this association study, we found that the CYP2D6*4 variant is a risk factor for PD in a Pakistani population that is known to be exposed to pesticides.. Both allele and genotype frequencies of CYP2D6*4 variant were significantly higher in the PD patients than in controls. We observed a 2.5 fold elevated risk of PD in the CYP2D6*4 carriers compared to non carriers which is in a similar range observed by other studies [9]. Importantly, there were no homozygous CYP2D6*4 carriers in the control group in contrast to 8 in the patient group suggesting that the absence enzyme function strongly increases the disease susceptibility in our study group. Heterozygous and homozygous CYP2D6*4 carriers had a younger age of PD onset of approximately 5 and 13 years, respectively, when compared to non-carriers and there was a trend to a more tremor-dominant disease. Agúndez et al., [32] reported a similar reduction in the age of PD onset in a Spanish population. In addition, the CYP2D6*4 allele was more frequent in patients with a tremor dominant disease in our study consistent with observations of Akhmedova et al., [33]. Our results are therefore in line with the previous studies.

A recent meta-analysis involving case–control studies questioned an association of CYP2D6*4 allele with PD risk in the Asian populations although an association was confirmed for the European populations in this study [9]. Of note, the Asian studies that were included in this meta-analysis were comparably small especially when considering the low frequency of the CYP2D6*4 allele in the Asian population. The frequency of CYP2D6*4 in our control group was estimated to be 4.4%. Naveen et al., and Adithan et al., [34, 35] reported CYP2D6*4 frequencies of 7.3% for south Indians and 6.6% for Tamilian populations, respectively. A study involving worldwide CYP2D6 genetic variation reported a CYP2D6*4 frequency of 8.1% (range 4–12%) for Central/South Asian ethnic groups mainly constituting Pakistani subpopulations [11]. The frequency of CYP2D6*4 among south Asians is therefore significantly lower as compared to some European populations, which was reported to be 17.2% (range 8.8–20.8%).

CYP2D6*4 allele carriers are poor metabolizers of potential environmental neurotoxins and it is possible that this is the cause for the increased susceptibility to PD. Low ORs in the range of 1.05 to 1.6 for homozygous CYP2D6*4 carriers were reported in several studies, which may be explained by a lack of exposure to the potential environmental neurotoxins in the investigated patient groups [9]. The PD cases and control individuals of our study were mainly from rural areas living near farmlands in tobacco growing areas. Such rural dwellings in Pakistan have been characterized as having a higher ambient air pollution (e.g. daily particulate above the world average), intensive use of agricultural pesticides and pesticide contamination of subsoil and ground sources of drinking water [25, 36–38]. Interestingly, previous studies found that the risk to develop PD increases between two to eight fold in homozygous CYP2D6*4 carriers depending on the level of the pesticide exposure [24, 39]. Together with our data these findings suggest that individuals with a poor metabolizer phenotype resulting from a non-functional debrisoquine 4-hydroxylase enzyme in CYP2D6*4 allele carriers or potentially also from other polymorphisms in the *CYP2D6* gene [39] exhibit an increased risk for PD especially when exposed to potential neurotoxins such as pesticides in the intensive agricultural landscapes.

Although potential pesticide toxicity is a strong candidate mechanism by which CYP2D6*4 allele may contribute to the PD risk there are alternate explanations that can be envisioned as the basis of strong association between PD and CYP2D6*4. The CYP2D6 enzyme is involved in the metabolism of a variety of endogenous compounds which may be affected in CYP2D6*4 carriers [40]. In addition apart from predominantly endoplasmic reticulum-targeted CYP2D6 mitochondrial targeting of CYP2D6 has also been described in the PD relevant dopaminergic neurons [41]. Since mitochondrial dysfunction is a known cellular change observed in PD, the lack of CYP2D6 enzyme activity in CYP2D6*4 allele carriers may contribute to the PD pathogenesis via this cellular compartment.

## Conclusion

In conclusion, we show that the CYP2D6*4 allele increases the PD risk in a Pakistani subpopulation with high prevalence of pesticide exposure. Further studies are warranted to identify the particular pesticide neurotoxins and the mechanism underlying the association between CYP2D6*4 allele and PD susceptibility in Pakistani and other populations.

## Abbreviations

CYP2D6*4: Cytochrome P450 2D6 allelic variant 4
GCRh38: Genome reference consortium (human) 38
OR: Odds ratio
PD: Parkinson’s disease
PIGD: Postural instability/gait difficulty
SD: Standard deviation
SPSS: Statistical package for the social sciences
TD: Tremor dominant
UPDRS: Unified Parkinson’s disease rating scale

## References

[1] Dauer W, Przedborski S (2003). Parkinson’ s disease: mechanisms and models. Neuron.

[2] Verstraeten A, Theuns J, Van Broeckhoven C (2015). Progress in unraveling the genetic etiology of Parkinson disease in a genomic era. Trends Genet.

[3] Cannon JR, Greenamyre JT (2013). Gene-environment interactions in Parkinson’s disease: specific evidence in humans and mammalian models. Neurobiol Dis.

[4] Evans WE, Relling MV (1999). Pharmacogenomics: translating functional genomics into rational therapeutics. Science (80-).

[5] Tang C, Prueksaritanont T (2010). Use of in vivo animal models to assess pharmacokinetic drug-drug interactions. Pharm Res.

[6] McNaught KSP, Carrupt PA, Altomare C, Cellamare S, Carotti A, Testa B (1998). Isoquinoline derivatives as endogenous neurotoxins in the aetiology of Parkinson’s disease. Biochem Pharmacol.

[7] Mann A, Miksys SL, Gaedigk A, Kish SJ, Mash DC, Tyndale RF (2012). The neuroprotective enzyme CYP2D6 increases in the brain with age and is lower in Parkinson’s disease patients. Neurobiol Aging.

[8] Christensen PM, Gotzsche PC, Brosen K (1998). The sparteine/debrisoquine (CYP2D6) oxidation polymorphism and the risk of Parkinson’s disease: a meta-analysis. Pharmacogenetics.

[9] Lu Y, Mo C, Zeng Z, Chen S, Xie Y, Peng Q (2013). CYP2D6*4 allele polymorphism increases the risk of Parkinson’s disease: evidence from meta-analysis. PLoS One.

[10] Persad AS, Stedeford T, Tanaka S, Chen L, Banasik M (2003). Parkinson’s disease and CYP2D6 polymorphism in Asian populations: a meta-analysis. Neuroepidemiology.

[11] Sistonen J, Sajantila A, Lao O, Corander J, Barbujani G, Fuselli S (2007). CYP2D6 worldwide genetic variation shows high frequency of altered activity variants and no continental structure. Pharmacogenet Genomics.

[12] Kanthasamy AG, Kitazawa M, Kanthasamy A, Anantharam V (2005). Dieldrin-induced neurotoxicity: relevance to Parkinson’s disease pathogenesis. Neurotoxicology.

[13] Linder L, City SL (2015). HHS Public Access.

[14] Dhillon AS, Tarbutton GL, Levin JL, Plotkin GM, Lowry LK, Nalbone JT (2008). Pesticide/environmental exposures and Parkinson’s disease in East Texas. J Agromedicine.

[15] Fleming L, Mann JB, Bean J, Briggle T, Sanchez-Ramos JR (1994). Parkinson’s disease and brain levels of organochlorine pesticides. Ann Neurol.

[16] Firestone JA, Smith-Weller T, Franklin G, Swanson P, Longstreth WT, Checkoway H (2005). Pesticides and risk of Parkinson disease: a population-based case–control study. Arch Neurol.

[17] Manthripragada AD, Costello S, Cockburn MG, Bronstein JM, Ritz B (2010). Paraoxonase 1, agricultural organophosphate exposure, and Parkinson disease. Epidemiology.

[18] Corrigan FM, Wienburg CL, Shore RF, Daniel SE, Mann D (2000). Organochlorine insecticides in substantia nigra in Parkinson’s disease. J Toxicol Environ Health A.

[19] Brown TP, Rumsby PC, Capleton AC, Rushton L, Levy LS (2006). Pesticides and Parkinson’s disease - Is there a link?. Environ Health Perspect.

[20] Mutch E, Williams FM (2006). Diazinon, chlorpyrifos and parathion are metabolised by multiple cytochromes P450 in human liver. Toxicology.

[21] Sams C, Mason HJ, Rawbone R (2000). Evidence for the activation of organophosphate pesticides by cytochromes P450 3A4 and 2D6 in human liver microsomes. Toxicol Lett.

[22] Mutch E, Daly AK, Leathart JBS, Blain PG, Williams FM (2003). Do multiple cytochrome P450 isoforms contribute to parathion metabolism in man?. Arch Toxicol.

[23] Costa LG, Giordano G, Guizzetti M, Vitalone A (2008). Neurotoxicity of pesticides: a brief review. Front Biosci.

[24] Elbaz A, Levecque C, Clavel J, Vidal J-S, Richard F, Amouyel P (2004). CYP2D6 polymorphism, pesticide exposure, and Parkinson’s disease. Ann Neurol.

[25] Tariq MI, Afzal S, Hussain I, Sultana N (2007). Pesticides exposure in Pakistan: a review. Environ Int.

[26] Hakeem, Khalid Rehman, Akhtar, Javaid, Sabir M, editor. Soil Science: Agricultural and Environmental Prospectives [Internet]. Springer International Publishing Switzerland; 2016. Available from: https://books.google.de/books?id=jCTFDAAAQBAJ.

[27] Ahad K, Mohammad A, Khan H, Ahmad I, Hayat Y (2010). Monitoring results for organochlorine pesticides in soil and water from selected obsolete pesticide stores in Pakistan. Environ Monit Assess.

[28] Khan DA, Bhatti MM, Khan FA, Naqvi ST, Karam A (2008). Adverse effects of pesticides residues on biochemical markers in pakistani tobacco farmers. Int J Clin Exp Med.

[29] Sambrook J, Fritsch EF, Maniatis T (1989). Molecular cloning: a laboratory manual.

[30] Jankovic J, McDermott M, Carter J, Gauthier S, Goetz C, Golbe L (1990). Variable expression of Parkinson’s disease: a base-line analysis of the DATATOP cohort. The Parkinson Study Group. Neurology.

[31] Chou WH, Yan FX, Robbins-Weilert DK, Ryder TB, Liu WW, Perbost C (2003). Comparison of two CYP2D6 genotyping methods and assessment of genotype-phenotype relationships. Clin Chem.

[32] Agundez JA, Jimenez-Jimenez FJ, Luengo A, Bernal ML, Molina JA, Ayuso L (1995). Association between the oxidative polymorphism and early onset of Parkinson’s disease. Clin Pharmacol Ther.

[33] Akhmedova SN, Pushnova EA, Yakimovsky AF, Avtonomov VV, Schwartz EI (1995). Frequency of a specific cytochrome P4502D6B (CYP2D6B) mutant allele in clinically differentiated groups of patients with Parkinson disease. Biochem Mol Med.

[34] Naveen AT, Adithan C, Soya SS, Gerard N, Krishnamoorthy R (2006). CYP2D6 genetic polymorphism in South Indian populations. Biol Pharm Bull.

[35] Adithan C, Gerard N, Vasu S, Rosemary J, Shashindran CH, Krishnamoorthy R (2003). Allele and genotype frequency of CYP2C19 in a Tamilian population. Br J Clin Pharmacol.

[36] Siddique N, Waheed S (2014). Source apportionment using reconstructed mass calculations. J Environ Sci Heal A Tox Hazard Subst Environ Eng.

[37] Azizullah A, Khattak MNK, Richter P, Häder DP (2011). Water pollution in Pakistan and its impact on public health - a review. Environ Int.

[38] Khan DA, Hashmi I, Mahjabeen W, Naqvi TA (2010). Monitoring health implications of pesticide exposure in factory workers in Pakistan. Environ Monit Assess.

[39] Tsuboi Y (2012). Environmental-Genetic Interactions in the Pathogenesis of Parkinson's Disease. Exp Neurobiol..

[40] Wang X, Li J, Dong G, Yue J (2014). The endogenous substrates of brain CYP2D. Eur J Pharmacol.

[41] Bajpai P, Sangar MC, Singh S, Tang W, Bansal S, Chowdhury G (2013). Metabolism of 1-methyl-4-phenyl-1,2,3/6-tetrahydropyridine by mitochondrion-targeted cytochrome P450 2D6 implications in parkinson disease. J Biol Chem.

